# The pattern and dynamics of white matter alterations in Spinocerebellar ataxia type 1: A diffusion-weighted magnetic resonance imaging study

**DOI:** 10.1016/j.nicl.2025.103783

**Published:** 2025-04-21

**Authors:** Kirsten C.J. Kapteijns, Teije H. van Prooije, Hao Li, Tom W.J. Scheenen, Anil Man Tuladhar, Bart P. van de Warrenburg

**Affiliations:** a Department of Neurology, Donders Institute for Brain, Cognition, and Behavior, Radboud University Medical Center, Netherlands; b Department of Medical Imaging, Radboud University Medical Center, Netherlands

**Keywords:** Spinocerebellar ataxia type 1, Diffusion, Longitudinal study, White matter integrity

## Abstract

**Background:**

Spinocerebellar ataxia type 1 (SCA1) is a rare, neurodegenerative disease. Upcoming clinical disease-modifying trials require biomarkers sensitive to disease progression. This study aims to investigate diffusion MRI (dMRI) metrics as a possible outcome measure in such trials.

**Methods:**

46 participants (26 SCA1, 20 matched healthy controls (HC)) underwent 3 T MRI examination and clinical assessment of ataxia severity (SARA) at three timepoints over the duration of two years, including dMRI. Diffusion metrics (fractional anisotropy, mean diffusivity, radial diffusivity, axial diffusivity) were examined using tract-based spatial statistics (TBSS) and ROI-based extraction. Results were evaluated for change over time and relation to disease severity.

**Results:**

Cerebellar white matter, in particular all cerebellar peduncles, showed significant (p < 0.001) differences between SCA1 and HC groups at baseline in all diffusion metrics. After two years, dynamics were only observed in the inferior cerebellar peduncle (ICP). However, a sub-group of early-stage disease patients (SARA ≤ 11) showed significant change in the corticospinal tract (CST) and pontine crossing tract (PCT), indicating stage-dependent dynamics. Cortical regions did not show cross-sectional differences between groups, but did change significantly in both anterior and posterior regions in the SCA1 group (p < 0.001).

**Conclusion:**

SCA1 patients showed significantly impaired white matter integrity in the cerebellar regions, when compared to HC. At the group level, diffusion metrics show dynamic effects in the ICP and in cortical regions. Patients in early disease stages furthermore show dynamic change in the CST and PCT. This indicates that white matter alterations follow a specific pattern throughout the disease and that measurements thereof are most useful in clinical trials targeting early disease stages.

## Highlights

• Significant differences in diffusion metrics between SCA1 and HC groups implies white matter impairment.
• The inferior cerebellar peduncle showed dynamics over a period of 2-years.
• A sub-group of early-stage SCA1 patients showed dynamics in multiple cerebellar regions.
• Cerebral changes in diffusivity occurred after 2-years in the SCA1 group only.

## 1 Introduction

Spinocerebellar ataxia type 1 (SCA1) is a currently incurable, genetic neurodegenerative disease affecting one to two individuals in 100,000 (Zoghbi and Orr, 1995). The disease clinically manifests as a progressive cerebellar ataxia, often with additional motor and non-motor symptoms. SCA1 is caused by an expansion of a CAG repeat in the *ATXN1* gene, leading to a polyglutamine stretch in the corresponding ataxin-1 protein (Zoghbi and Orr, 1995). In comparison to other SCA’s, the rate of progression in SCA1 is relatively high (Diallo et al., 2018; Jacobi et al., 2015).

SCA1 is mainly characterized by neuronal degeneration. Previous studies have shown significant reductions of brain volume in patients with SCA1, especially of the cerebellum and brainstem (Guerrini et al., 2004, Adanyeguh et al., 2018, Deelchand et al., 2019, Nigri et al., 2022, Chandrasekaran et al., 2023, van Prooije et al., 2024). One prominent volume significantly decreasing over time is the cerebellar white matter. This has led to an increasing interest in white matter alterations as possible sensitive progression markers in SCA1 (Mandelli et al., 2007, Della Nave et al., 2008, Martins Junior et al., 2018, Park et al., 2020). Diffusion-weighted magnetic resonance imaging (dMRI) is a validated method for investigating white matter microstructure in-vivo. WM integrity can be assessed through the dMRI measures fractional anisotropy (FA), mean diffusivity (MD), axial diffusivity (AD), and radial diffusivity (RD). Although dMRI research in SCA1 is relatively limited, several cross-sectional studies report significant differences in areas such as the brainstem and cerebellum when comparing SCA patients to healthy controls (Kang et al., 2014, Martins Junior et al., 2018, Prakash et al., 2009, Ye et al., 2024). One longitudinal study into multiple SCA types by Park et al. (2020), used longitudinal data of nine SCA1 patients to investigate potential imaging biomarkers for disease severity and progression. They identified AD in the middle cerebellar peduncle (MCP) as a candidate marker of disease progression. Moreover, Rezende et al. (2024) included data of ten SCA1 patients with a six-month follow-up and showed high responsiveness to change in microstructural measures from the MCP and the right corona radiata, further supporting the possibility of utilizing these measures as candidate markers for tracking disease progression. More research using larger samples of SCA1 patients, including patients with a higher disease severity, is needed to more comprehensively evaluate dMRI as a surrogate marker.

We performed a larger, multi-timepoint longitudinal dMRI study in a Dutch SCA1 cohort in order to explore whether dMRI can provide imaging biomarkers that can serve as outcome measures in disease-modifying clinical trials, and to gain more insight in WM alterations in SCA1. We utilized cross-sectional and longitudinal analyses, both data-driven as well as hypothesis-based, through the use of specific regions of interest, cross-correlating dMRI findings with clinical measures of disease severity.

## 2 Methods

### 2.1 Participants and study design

This study included twenty-six Dutch SCA1 carriers and twenty age- and gender-matched healthy controls (HC). The HC group consisted both of relatives of patients with a negative test for SCA1 and unrelated volunteers. The study was conducted according to the principles of the Declaration of Helsinki and was approved by the Local Ethics Committee (CMO-2019–5377). Written informed consent was obtained for all participants. Participants underwent a 3.0 T MRI scan at three time points, with a year (52 ± 2 weeks) between visits.

### 2.2 Clinical assessment of disease severity

All participants underwent a neurological assessment by the same trained assessor at all three timepoints. Ataxia severity was assessed using the scale for the assessment and rating of ataxia (SARA), the reference standard for rating SCA disease severity (Schmitz-Hubsch et al., 2006). The scale consists out of 8 sub-items providing a score from 0 to 40, with higher scores indicating higher disease severity. SCA1 mutation carriers were classified as ataxic if their SARA score was ≥ 3. SCA1 patients self-reported their age-of-onset, which was used to calculate their disease duration.

The SCA1 group was further divided into subgroups. Groups were based on the median of the whole group to create groups of approximately equal size: SCA_early_ (≤11) and SCA_late_ (>11) disease state subgroups based on the median SARA score at baseline. These subgroups allow examining correlations between dMRI changes and clinical indices of severity.

### 2.3 Image aquisition

All MRI examinations were performed on a 3.0 T MRI scanner (Magnetom Prisma-fit, Siemens Healthineers, Erlangen). The protocol included an anatomical 3D T1-weighted magnetization-prepared rapid gradient echo (MPRAGE) sequence (TR = 2300 ms/TE = 4.4 ms/TI = 950 ms; voxel size = 0.9 mm isotropic) and a spin-echo multiband diffusion MRI sequence (TR = 2700 ms/TE = 73 ms; voxel size = 1.9 mm isotropic; 64 diffusion directions; b-values 0, 1000 and 3000 s/mm^2^). The b0 image was repeated with opposing phase encoding in the anterior-posterior direction for susceptibility-induced distortion corrections using ‘top-up’ function in FSL.

### 2.4 Image processing

All scans were visually inspected and pseudonymized for further processing. The diffusion data was pre-processed to remove Gibbs ringing artifacts, correct for head motion, eddy current distortions, susceptibility-induced distortion (top-up function in FSL), and intensity bias using MRtrix 3.0 (Tournier et al., 2019), FSL 6.0.6 (Jenkinson et al., 2012) and Advanced Normalization Tools (ANTs) 2.1.0 (Avants et al., 2011). For the data-driven analysis, we estimated whole-brain maps of FA, AD, MD, and RD from the pre-processed dMRI data using the ‘dtifit’ function within FSL (Behrens et al., 2003, Jbabdi et al., 2012) as earlier described (Li et al., 2024).

The TBSS package was used for further processing and analysis (Smith et al., 2006). A mean FA image was formed and thinned with a threshold of > 0.2 to create a mean FA skeleton while accounting for inter-subject variability. The FA data was skeletonized and utilised for further voxel-wise statistics by projection onto this skeleton. Skeletonized images for mean diffusivity (MD), radial diffusivity (RD), and axial diffusivity (AD) were created with the registrations calculated for the FA maps.

Binary masks were created for regions of interest (ROIs): the superior, inferior, and middle cerebellar peduncles (SCP; ICP; MCP), pontine crossing tract (PCT), and corticospinal tracts (CST), using the JHU WM DTI-based brain white matter anatomy atlas(Hua et al., 2008). These regions were chosen for the hypothesis-based analysis as they are known to be affected in SCA1 (van Prooije et al., 2024). The masks could then be used to extract the patient-specific dMRI metric means within each ROI. This enabled individual analysis and correlations with clinical scores.

### 2.5 Statistical analysis

#### 2.5.1 Cross-sectional

To assess group differences between SCA1 and HC at baseline, we used t-tests or χ2 tests as appropriate. Wilcoxon rank sum tests were used for non-Gaussian distributed variables. To evaluate voxel-based diffusion measures (i.e., FA, MD, RD, AD) differences between the HC and SCA1 groups, the generalized linear model (GLM) with p < 0.05 as a significant threshold was employed using the randomise tool (FSL 6.0.5) with 5000 permutations. The threshold-free cluster enhancement (TFCE) based family-wise error correction (FWE) approach was employed to correct multiple comparisons and identify the significantly differing clusters between the SCA1 and HC group at baseline. Age and sex were used as confounding variables within each generalized linear model.

We applied GLMs to analyse diffusion measures of the extracted ROIs and compare SCA1 patients to the HC, using R. Age and sex were added to the model as covariates. To analyse correlations between diffusion measures and disease severity, we used Pearson’s or Spearman correlation coefficients using a linear regression model including age and sex as covariates. *P*-values are adjusted using a Holm-Bonferroni correction to account for multiple testing. A *p* value of < 0.05 was considered significant.

#### 2.5.2 Longitudinal

FSL’s randomise tool for repeated measures (ANOVA) was used to assess significant differences between time points within the SCA1 group for each dMRI metric, with 5000 permutations and TFCE.

All three timepoints were extracted for individual ROIs with significant differences between SCA1 and HC at baseline. We used a mixed-effects model with the specific regional dMRI measure as the dependent variable and a categorical visit variable as a fixed-effect. Age and sex were used as confounders in the model, as well as an random intercept for family. Standardized beta coefficients were extracted to enable comparison between the different dMRI measures. In order to evaluate stage-specific changes over time, we divided the SCA1 group in early- and late-stage subgroups as mentioned. These groups were all compared with the HC group.

Holm-Bonferroni corrected p-values are reported, with a p < 0.05 significance threshold.

We used R studio (version 4.1.3;2022) to analyse the data (Team., 2021). The package *lme4* was used for linear mixed-effects modelling (Bates et al., 2015) with sex and age as standard confounders.

## 3 Results

### 3.1 Participant characteristics

Twenty-six ataxic SCA1 patients and twenty-one healthy controls were included in this study. Twenty-two SCA1 patients and nineteen healthy controls completed the longitudinal parts of the study (87 %; Supplemental S1). One participant remained below the ataxic SARA threshold of 3 at all timepoints and was categorized as pre-ataxic. We excluded this participant from further group analyses. Group characteristics are summarized in Table 1.

**Table 1:** Characteristics at baseline of healthy controls (HC) and SCA1 patients [mean].

	HC	SCA1	SCA1_early_	SCA1_late_
N (% female)	21 (43 %)	26 (42 %)	12 (42 %)	14 (46 %)
Age (SD)	50.1 (±13.4)	50.1 (±13.6)	45.6 (±13.2)	54.7 (±13.0)
Age of onset (SD)	N/A	44.0 (±10.9)	42.1 (±13.0)	45.2 (±9.6)
Disease duration (range)	N/A	6.4 (0 – 20)	3.2 (0 – 10)	9.3 (2 – 20)
Expanded CAG repeat	N/A	45.8 (±5.3)	46.1(±5.2)	45.1(±5.5)
SARA (range)	0.4 (0 – 2)	12.0 (1 – 29.5)***	7.5 (1 – 11)***	15.9 (11.5 – 29.5)***
SARA at 1Y follow-up	0.1 (0 – 1)	14.0 (2 – 32) ***	8.8 (2 – 14)***	18.4 (9.5 – 32) ***
SARA at 2Y follow-up	0.3 (0 – 2.5)	15.5 (3.5 – 31) ***	11.2 (3.5 – 17.5) ***	19.5 (11 – 31) ***

Between group means and differences (SCA1 vs HC; SCA1_early_ vs HC; SCA1_late_ vs HC), * p-value < 0.05, ** p-value < 0.005, *** p-value < 0.0005.

### 3.2 Cross-sectional results

**Fig. 1:**
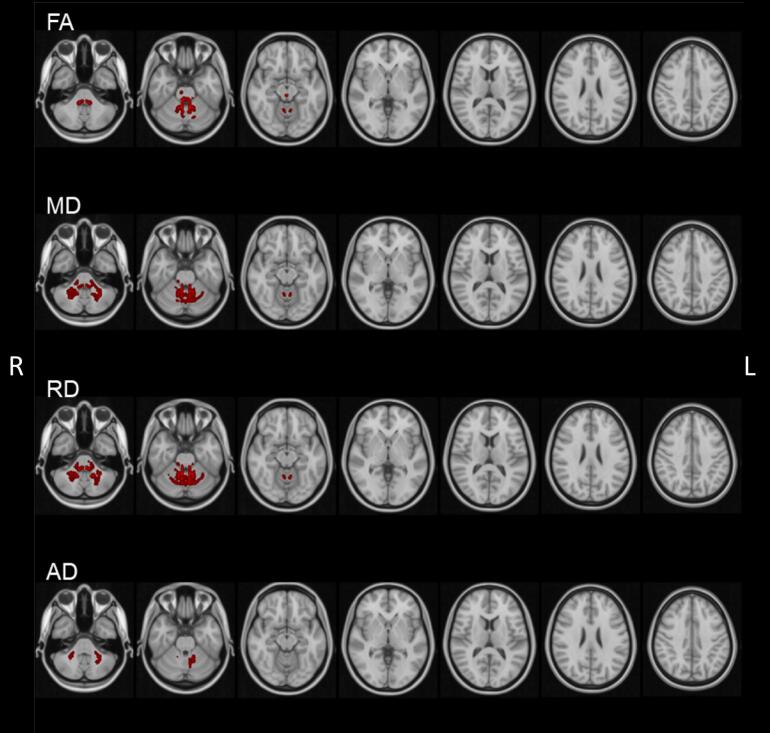
Group level tract-based spatial statistics (TBSS) results at baseline. Threshold-free cluster enhancement (TFCE) p < 0.001. Coloured areas indicate significant differences between healthy controls (HC) and SCA1 group. SCA1 showed significantly lower FA values in the cerebellum, while the remaining diffusivity values were higher in the cerebellum of SCA1 patients, compared to HC.

Fig. 1 illustrates the findings of our whole-brain TBSS analysis with group comparison between the SCA1 versus the HC group. The SCA1 group showed a significantly lower FA in the cerebellar area, compared to healthy controls. Significantly higher MD and RD values were found in the cerebellar white matter, with no involvement of cerebral cortical clusters in the SCA1 group. AD values also were significantly higher in several cerebellar white matter areas for the SCA1 group, although they were less pronounced compared to the other parameters.

**Fig. 2:**
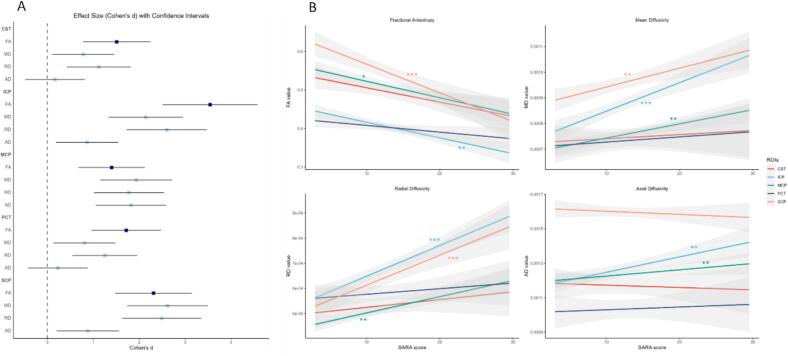
Cross-sectional ROI analysis. (A) Effect sizes (Cohen’s d) for each ROI diffusion measure comparison between HC and SCA1 group. Darkblue values (FA) show flipped to enable easier comparison between markers. With the exception of AD values in the CST and PCT, all values were significantly different between groups. (B) Visualization of the correlation between baseline ROI diffusion measures and baseline disease severity as measured by SARA. Significant correlations after Holm-Bonferroni correction are denoted with *p < 0.05, ** p < 0.01, *** p < 0.001. Abbreviations: Superior cerebellar peduncle (SCP), middle cerebellar peduncle (MCP), inferior cerebellar peduncle (ICP), pontine crossing tract (PCT), corticospinal tract (CST), fractional anisotropy (FA), mean diffusivity (MD), radial diffusivity (RD), axial diffusivity (AD).

ROI-specific diffusion parameters (i.e., FA, MD, RD, AD) showed significant differences between the two groups at baseline (Fig. 2; Supplemental S2). All regions of interest showed a significantly lower FA in the SCA1 group as compared to HC, indicating widespread diffusivity alterations. MD and RD were significantly decreased in all ROIs. Finally, AD significantly increased in all three of the cerebellar peduncles, but did not show differences in the PCT and CST. Cohen’s *d* was calculated for each HC-SCA1 pair to compare strengths of the found differences (Fig. 2). The diffusivity of cerebellar peduncles showed significant correlations with SARA in multiple dMRI measures (Supplemental S2). This relation was negative for the FA values, and positive for the MD, AD, and RD metrics, suggesting an increase of dMRI abnormalities with disease progression.

### 3.3 Longitudinal results

**Fig. 3:**
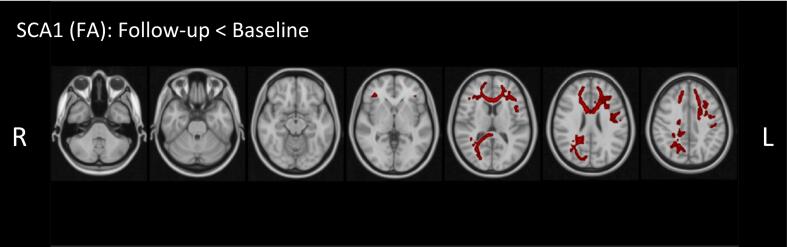
Tract-based spatial statistics (TBSS) FA results SCA1 baseline vs. SCA1 two-year follow-up. (p < 0.001, FWE- corrected for multiple comparisons using Threshold-Free Cluster Enhancement (TFCE)). FA values decreased in cortical areas, including the genu corpus callosum and corona radiata. TBSS results of HC baseline vs. HC two-year follow-up showed no significant clusters.

Whole-brain TBSS comparing the SCA1 baseline to the 2Y follow-up illustrated significant changes in both frontal and posterior cortical brain areas in the SCA1 group (Fig. 3). FA significantly decreased, whereas MD and RD increased after two years in cerebral brain areas. These changes were not observed in similar whole-brain TBSS baseline versus 2Y follow-up analysis in the HC group. Neither the SCA1 nor HC group showed significantly changed clusters in any of the diffusion metrics within the cerebellar areas of the brain during the 2Y follow-up whole-brain comparison.

**Fig. 4:**
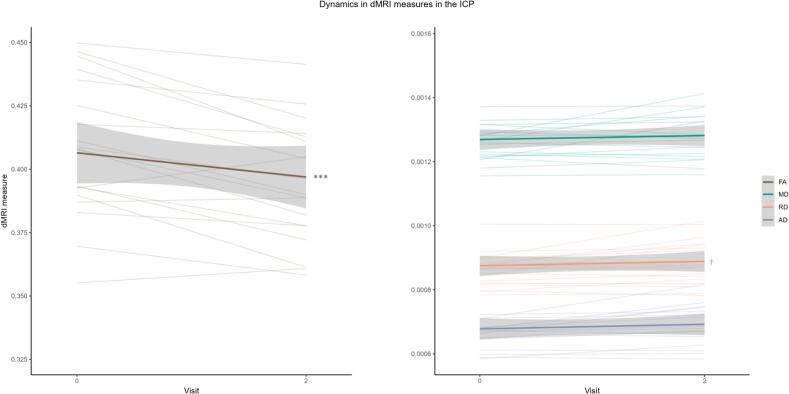
Diffusivity measures in the ICP over time. Significance is shown for the change over time corrected for confounders age and sex. Significant changes after Holm-Bonferroni correction or correlations are denoted with ^†^p < 0.07, *p < 0.05, ** p < 0.01, *** p < 0.001.

Longitudinal analysis of the ROIs with a significant correlation with SARA at baseline, showed significant change at both one year and two year follow up of the ICP FA (β_Y1_ = −0.60, p < 0.001; β_Y2_ = −0.51, p < 0.003) (Fig. 4). ICP MD and RD showed significant change after one year (MD: β_Y1_ = −0.65, p < 0.01; RD: β_Y1_ = −0.62, p < 0.001), but not after two years.

More explorative longitudinal analysis of the SCA1 early and late subgroup illustrates possible stage-dependent change. Within the SCA1_early_ group significant changes were observed in the CST at 2Y follow-up (MD β_Y2_ = -0.66, p < 0.02) and PCT (MD: β_Y2_ = -0.97, p < 0.01; RD: β_Y2_ = -0.93, p < 0.05). These changes were not observed when comparing the SCA1_late_ to the HC group (Supplemental S5-S6). This implies dynamic white matter pathology in specific areas in the early disease stage.

## 4 Discussion

This study investigated cross-sectional and longitudinal alterations in white matter integrity in a relatively large, single-center cohort of symptomatic SCA1 patients, over two years. Our findings confirm earlier studies (Park et al., 2020, Rezende et al., 2024), highlighting the discriminative value of MRI diffusion measures, and expand knowledge on white matter alterations as part of the pathophysiology in SCA1. Importantly, our findings indicate a use for specific dMRI measures as outcome measures in short (≤2 years) clinical trials.

Cerebellar white matter has been shown to be affected in SCA1 in earlier studies (Adanyeguh et al., 2018, Deelchand et al., 2019, Nigri et al., 2022, van Prooije et al., 2024). It is consistently shown that cerebellar white matter deteriorates over time, even in relatively short follow-ups (6 months – 1 year). We provide further insight into these white matter changes in SCA1, as we observed a series of abnormal diffusion metrics. First, significantly lower FA, and higher MD, AD, and RD values were observed in all cerebellar peduncles. Secondly, higher MD was localized to the cerebellum, whereas a higher RD was found also in the cerebral cortical regions. This might suggest that, at least for cerebral involvement, specific white matter changes (‘demyelination’) precede neuronal degeneration. These findings furthermore support the recent notion of oligodendrocyte involvement in the pathogenesis of specific SCAs, as a possible explanation for impaired myelination (Klawiter et al., 2011, Putka et al., 2023). Combined, these data suggest that white matter changes are an important component of the disease process in SCA1.

We identified several areas in the cerebellar white matter, particularly the cerebellar peduncles, in which diffusion measures differentiate SCA1 patients from controls and correlate with disease severity. However, our longitudinal analysis showed an absence of dynamic changes over time for most of these differentiating dMRI metrics over an interval of two years. The exception was the ICP, of which FA values showed robust change at both timepoints, suggesting that dMRI metrics within the cerebellum have promising utility as a surrogate outcome measure in clinical trials with durations of two years or less for the entire symptomatic stage.

The limited dynamics at first glance contradict earlier findings by Park et al. (2020) and Rezende et al. (2024), both suggesting that several dMRI metrics are possible biomarkers of disease progression in SCA1. However, their SCA1 cohorts had a significantly lower SARA mean, and their pre-symptomatic converters already exhibited WM alterations. To explore the disease stages, and come closer to the previously investigated cohorts, we divided out SCA1 group in the early and late subgroups, to evaluate if dMRI metrics could be biomarkers at specific disease stages. In our subgroup of SCA1 patients with a baseline SARA lower than 11, we observed multiple dMRI metrics with significant changes over time, whereas these changes were not observed in the SCA1_late_ group. This might indicate that the changes in WM microstructure at the level of cerebellum and brainstem emerge during the pre-symptomatic and early stage of SCA1, while their rate of change is slowing down as the disease progresses. As our cross-sectional data showed a strong correlation of dMRI changes with disease severity, there is still an indication that WM microstructure deteriorates over the whole symptomatic phase of SCA1. However, the rate of this deterioration in later symptomatic stages might be low and therefore not measurable longitudinally over our relatively short study period of two years.

Interestingly, we did observe significant longitudinal changes in cerebral cortical diffusion measures in the SCA1 group through whole-brain analysis. These clusters were not found in the HC group, suggesting these changes to be disease specific. Alterations of WM microstructure in the cortical areas potentially manifest during the later stages of the disease, which are well represented in our symptomatic SCA1 cohort. A previous post-mortem study in several SCA subtypes illustrated loss of fibres in the frontal regions of the brain (Rüb et al., 2012). Whether the involvement of these cortical areas has a clinical correlate, for example, on cognitive function or as collapse of compensatory mechanisms, remains speculative and requires further study.

Although this is the largest, single-center longitudinal dMRI study in a symptomatic SCA1 cohort, our sample size is still limited and our cohort lacks presymptomatic carriers. Larger studies, which will need to be international multicenter studies, and the aggregation of currently available dMRI data is needed to validate our findings and more robustly map the dynamics of WM microstructure alterations across the various disease stages in SCA1.

## 5 Conclusion

In this study, we demonstrated significant alterations in cerebellar WM microstructure in symptomatic SCA1 patients compared to healthy controls, in particular in cerebellar peduncles and brainstem, indicating WM pathology as part of the disease process in SCA1. These alterations correlated with disease severity, but showed little sensitivity to change over a two-year interval in the known-to-be-affected areas, except for ICP alterations. Instead, whole-brain analysis show microstructural changes emerging at the cerebral cortical regions over time. In addition, our data suggest stage-dependent WM alterations, where CST and PCT tracts only show changes in a subgroup of early stage SCA1 patients.

## Funding

This work was supported by a grant from ZonMw (40-44600-98-606).

## Declaration of Competing Interest

The authors declare that they have no known competing financial interests or personal relationships that could have appeared to influence the work reported in this paper.

## Data Availability

Data will be made available on request.
