# Supplementary material for: The pattern and dynamics of white matter alterations in Spinocerebellar ataxia type 1: A diffusion-weighted magnetic resonance imaging study
Source: Neuroimage Clin. 2025 Apr 21;46:103783. doi: 10.1016/j.nicl.2025.103783 (PMC12264234; doi:10.1016/j.nicl.2025.103783)
Supplement: Supplementary Data 1 [file mmc1.docx]

**Supplementary materials**

**Figure S1.** Flow chart of SCA1 mutation carriers and healthy controls inclusion at baseline, follow-up after one year, and follow-up after two years.


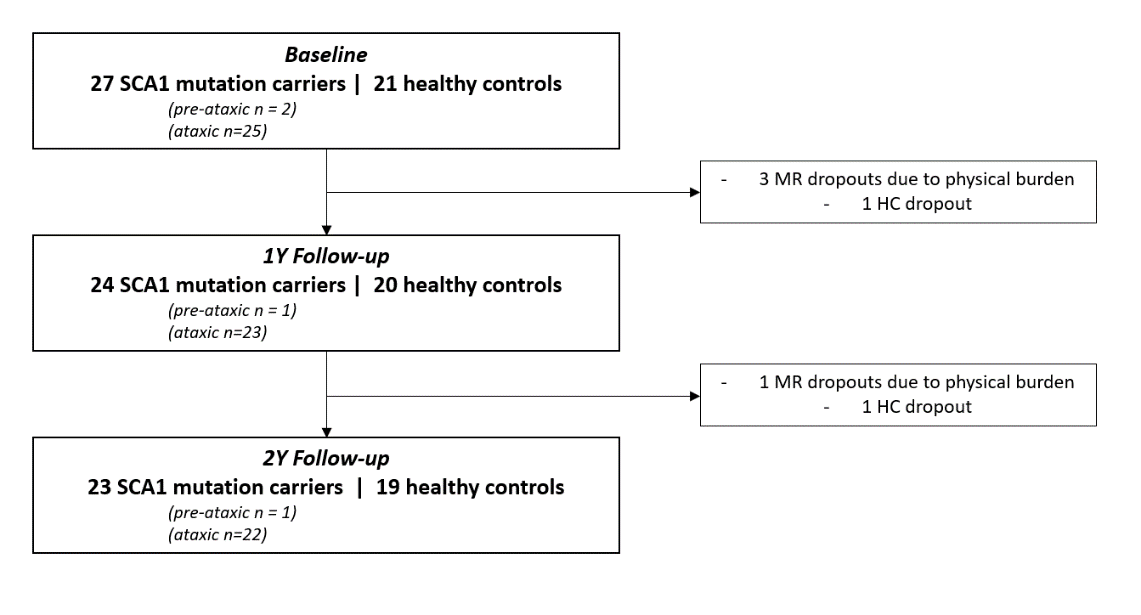


**Table S2:** Baseline diffusivity measures of SCA1 patients and healthy controls (HC). Where necessary left and right values were averaged. Significant differences after Holm-Bonferroni. Abbreviations: Superior cerebellar peduncle (SCP), middle cerebellar peduncle (MCP), inferior cerebellar peduncle (ICP), pontine crossing tract (PCT), corticospinal tract (CST), fractional anisotropy (FA), mean diffusivity (MD), radial diffusivity (RD), axial diffusivity (AD).

| **ROI** | **dMRI measure** | **HC mean** | **HC SD** | **SCA1 mean** | **SCA1 SD** | **% difference** | **p-value** |
| --- | --- | --- | --- | --- | --- | --- | --- |
| MCP | FA | 0.555 | 0.018 | 0.511 | 0.033 | -8.0 | **0.000** |
|  | MD | 0.00068 | 0.00002 | 0.00076 | 0.00005 | 11.8 | **0.000** |
|  | RD | 0.00044 | 0.00002 | 0.00052 | 0.00005 | 17.9 | **0.000** |
|  | AD | 0.00115 | 0.00003 | 0.00123 | 0.00005 | 7.1 | **0.000** |
| ICP | FA | 0.508 | 0.030 | 0.399 | 0.028 | -21.3 | **0.000** |
|  | MD | 0.00075 | 0.00003 | 0.00089 | 0.00007 | 19.6 | **0.000** |
|  | RD | 0.00051 | 0.00003 | 0.00069 | 0.00008 | 35.2 | **0.000** |
|  | AD | 0.00121 | 0.00004 | 0.00129 | 0.00008 | 6.3 | **0.000** |
| SCP | FA | 0.663 | 0.029 | 0.545 | 0.058 | -17.8 | **0.000** |
|  | MD | 0.00081 | 0.00003 | 0.00096 | 0.00007 | 18.3 | **0.000** |
|  | RD | 0.00045 | 0.00004 | 0.00065 | 0.00010 | 44.9 | **0.000** |
|  | AD | 0.00155 | 0.00004 | 0.00159 | 0.00005 | 2.8 | **0.000** |
| CST | FA | 0.548 | 0.028 | 0.498 | 0.036 | -9.1 | **0.000** |
|  | MD | 0.00070 | 0.00003 | 0.00072 | 0.00006 | 3.7 | **0.046** |
|  | RD | 0.00047 | 0.00003 | 0.00052 | 0.00006 | 10.1 | **0.000** |
|  | AD | 0.00116 | 0.00005 | 0.00114 | 0.00008 | -1.4 | 0.066 |
| PCT | FA | 0.454 | 0.028 | 0.411 | 0.027 | -9.5 | **0.000** |
|  | MD | 0.00068 | 0.00004 | 0.00071 | 0.00006 | 4.9 | **0.003** |
|  | RD | 0.00051 | 0.00004 | 0.00056 | 0.00006 | 10.8 | **0.000** |
|  | AD | 0.00101 | 0.00005 | 0.00100 | 0.00009 | -1.0 | 0.085 |

**Table S3**: model outcomes for cross-sectional relationship between ROI dMRI measure and disease severity (measured by SARA score) at baseline. For the correlation with SARA only the SCA1 cohort was included in the analysis. p-value is Holm-Bonferonni corrected for multiple comparisons.

| **ROI** | **dMRI measure** | **Standardized Beta coefficient** | **p-value** | **CI lower** | **CI_upper** |
| --- | --- | --- | --- | --- | --- |
| MCP | FA | -0.628 | **0.011** | -1.012 | -0.244 |
|  | MD | 0.629 | **0.002** | 0.318 | 0.940 |
|  | RD | 0.654 | **0.002** | 0.328 | 0.979 |
|  | AD | 0.569 | **0.008** | 0.222 | 0.916 |
| ICP | FA | -0.756 | **0.001** | -1.118 | -0.394 |
|  | MD | 0.771 | **0.000** | 0.470 | 1.072 |
|  | RD | 0.835 | **0.000** | 0.550 | 1.120 |
|  | AD | 0.614 | **0.006** | 0.270 | 0.958 |
| SCP | FA | -0.773 | **0.000** | -1.044 | -0.502 |
|  | MD | 0.586 | **0.008** | 0.245 | 0.926 |
|  | RD | 0.730 | **0.000** | 0.436 | 1.023 |
|  | AD | -0.157 | 1.000 | -0.570 | 0.256 |
| CST | FA | -0.390 | 0.442 | -0.877 | 0.098 |
|  | MD | -0.002 | 1.000 | -0.432 | 0.428 |
|  | RD | 0.064 | 1.000 | -0.389 | 0.517 |
|  | AD | -0.083 | 1.000 | -0.501 | 0.336 |
| PCT | FA | -0.165 | 1.000 | -0.759 | 0.430 |
|  | MD | 0.038 | 1.000 | -0.392 | 0.469 |
|  | RD | 0.037 | 1.000 | -0.409 | 0.482 |
|  | AD | 0.039 | 1.000 | -0.382 | 0.460 |

**Table S4**: model outcome measures for longitudinal change in each ROI dMRI measure for the SCA1 group, controlled for age, sex, and family clustering. P-values are Holm-Bonferonni corrected for multiple comparisons.

| **ROI** | **Marker** | **Standardized β** | **SE** | ***p*** | **Standardized β** | **SE** | ***p*** |
| --- | --- | --- | --- | --- | --- | --- | --- |
| MCP | FA | -0.163 | 0.066 | 0.304 | -0.175 | 0.096 | 0.760 |
|  | MD | 0.176 | 0.068 | 0.278 | 0.053 | 0.093 | 1.000 |
|  | RD | 0.176 | 0.064 | 0.221 | 0.083 | 0.095 | 1.000 |
|  | AD | 0.140 | 0.087 | 1.000 | -0.018 | 0.095 | 1.000 |
| ICP | **FA** | **-0.601** | **0.098** | **0.000** | **-0.505** | **0.104** | **0.003** |
|  | MD | **0.649** | **0.124** | **0.002** | 0.309 | 0.119 | 0.278 |
|  | RD | **0.619** | **0.103** | **0.000** | 0.331 | 0.104 | 0.096 |
|  | AD | 0.652 | 0.173 | 0.033 | 0.240 | 0.152 | 1.000 |
| SCP | FA | -0.083 | 0.037 | 0.467 | -0.182 | 0.057 | 0.096 |
|  | MD | 0.177 | 0.082 | 0.467 | -0.008 | 0.117 | 1.000 |
|  | RD | 0.148 | 0.048 | 0.119 | 0.107 | 0.078 | 1.000 |
|  | AD | 0.165 | 0.192 | 1.000 | -0.482 | 0.220 | 0.467 |

**Table S5-S6:** model outcome measures for longitudinal change in each ROI dMRI measure for the SCA1 subgroups, controlled age, sex, and family clustering. P-values are Holm-Bonferonni corrected for multiple comparisons.

***S5: Early subgroup***

|  |  | **Y1** |  |  | **Y2** |  |  |
| --- | --- | --- | --- | --- | --- | --- | --- |
| **ROI** | **Marker** | **Standardized β** | **SE** | ***p*** | **Standardized β** | **SE** | ***p*** |
| MCP | FA | -0.199 | 0.133 | 1.000 | -0.196 | 0.173 | 1.000 |
|  | MD | 0.143 | 0.126 | 1.000 | -0.028 | 0.165 | 1.000 |
|  | RD | 0.191 | 0.114 | 1.000 | 0.016 | 0.196 | 1.000 |
|  | AD | 0.046 | 0.155 | 1.000 | -0.082 | 0.107 | 1.000 |
| ICP | FA | -0.862 | 0.217 | 0.107 | -0.713 | 0.193 | 0.198 |
|  | MD | 0.743 | 0.177 | 0.091 | 0.217 | 0.173 | 1.000 |
|  | RD | **0.850** | **0.162** | **0.023** | 0.358 | 0.175 | 1.000 |
|  | AD | 0.470 | 0.236 | 1.000 | -0.034 | 0.181 | 1.000 |
| SCP | FA | -0.081 | 0.076 | 1.000 | -0.173 | 0.124 | 1.000 |
|  | MD | 0.148 | 0.161 | 1.000 | -0.139 | 0.181 | 1.000 |
|  | RD | 0.137 | 0.090 | 1.000 | 0.045 | 0.132 | 1.000 |
|  | AD | 0.102 | 0.335 | 1.000 | -0.636 | 0.320 | 1.000 |
| CST | FA | 0.031 | 0.171 | 1.000 | -0.136 | 0.236 | 1.000 |
|  | MD | -0.256 | 0.165 | 1.000 | **-0.660** | **0.124** | **0.014** |
|  | RD | -0.171 | 0.170 | 1.000 | -0.408 | 0.152 | 0.804 |
| PCT | FA | 0.165 | 0.168 | 1.000 | 0.821 | 0.263 | 0.356 |
|  | MD | -0.167 | 0.146 | 1.000 | **-0.970** | **0.157** | **0.008** |
|  | RD | -0.194 | 0.149 | 1.000 | **-0.933** | **0.191** | **0.033** |

***S6: Late subgroup***

|  |  | **Y1** |  |  | **Y2** |  |  |
| --- | --- | --- | --- | --- | --- | --- | --- |
| **ROI** | **Marker** | **Standardized β** | **SE** | **p** | **Standardized β** | **SE** | **p** |
| MCP | FA | -0.284 | 0.146 | 1.000 | -0.315 | 0.214 | 1.000 |
|  | MD | 0.320 | 0.133 | 1.000 | 0.133 | 0.186 | 1.000 |
|  | RD | 0.317 | 0.139 | 1.000 | 0.183 | 0.198 | 1.000 |
|  | AD | 0.256 | 0.128 | 1.000 | 0.023 | 0.174 | 1.000 |
| ICP | FA | **-0.635** | **0.116** | **0.021** | -0.570 | 0.182 | 0.386 |
|  | MD | 0.812 | 0.196 | 0.107 | 0.473 | 0.182 | 0.895 |
|  | RD | 0.730 | 0.176 | 0.107 | 0.448 | 0.166 | 0.791 |
|  | AD | 0.912 | 0.231 | 0.119 | 0.493 | 0.209 | 1.000 |
| SCP | FA | -0.110 | 0.050 | 1.000 | -0.251 | 0.070 | 0.204 |
|  | MD | 0.241 | 0.103 | 1.000 | 0.116 | 0.176 | 1.000 |
|  | RD | 0.197 | 0.070 | 0.752 | 0.191 | 0.120 | 1.000 |
|  | AD | 0.253 | 0.196 | 1.000 | -0.285 | 0.312 | 1.000 |
| CST | FA | -0.215 | 0.103 | 1.000 | -0.271 | 0.202 | 1.000 |
|  | MD | -0.120 | 0.179 | 1.000 | -0.179 | 0.245 | 1.000 |
|  | RD | -0.026 | 0.160 | 1.000 | -0.064 | 0.242 | 1.000 |
| PCT | FA | -0.066 | 0.181 | 1.000 | 0.487 | 0.261 | 1.000 |
|  | MD | -0.017 | 0.167 | 1.000 | -0.327 | 0.194 | 1.000 |
|  | RD | -0.004 | 0.184 | 1.000 | -0.411 | 0.233 | 1.000 |
